# The use of non-bronchoscopic brushings to study the paediatric airway

**DOI:** 10.1186/1465-9921-6-53

**Published:** 2005-06-08

**Authors:** Catherine Lane, Scott Burgess, Anthony Kicic, Darryl Knight, Stephen Stick

**Affiliations:** 1 School of Paediatrics and Child Health, University of Western Australia, Nedlands, 6009, Western Australia, Australia; 2 Department of Respiratory Medicine, Princess Margaret Hospital for Children, Perth, 6001, Western Australia, Australia; 3 Department of Pharmacology and Therapeutics, University of British Columbia, Vancouver, V6Z 1Y6, British Columbia, Canada

## Abstract

**Background:**

The use of cytology brushes for the purpose of obtaining respiratory cells from adults for clinical and research purposes is well established. However, the safety and utility of non-bronchoscopic brushings to study the paediatric airway has not been assessed. The purpose of this study was to assess the practicality of using non-bronchoscopic brushing to sample epithelial cells from children for investigation of epithelial function in health and disease using a wide range of molecular and cellular techniques.

**Methods:**

Non-bronchoscopic brushing was investigated in a non-selected cohort of healthy, and mildly asthmatic children presenting for surgery unrelated to respiratory conditions, at the major children's hospital in Perth. Safety and side-effects of the procedure were assessed. Cell number, phenotype and viability were measured for all samples. The potential of these cells for use in long-term cell culture, immunohistochemistry, western blotting, quantitative PCR and gene arraying was examined.

**Results:**

Non-bronchoscopic brushing was well tolerated in all children. The only significant side effect following the procedure was cough: nursing staff reported cough in 20% of patients; parents reported cough in 40% of patients. Cells sampled were of sufficient quantity and quality to allow cell culture in 93% of samples. Similarly, protein and RNA extracted from the cells was suitable for investigation of both gene and protein expression using micro-array and real-time PCR.

**Conclusion:**

Non-bronchoscopic brushing in children is safe and easy to perform, and is not associated with any complications. Using this technique, adequate numbers of epithelial cells can be retrieved to allow cell culture, western blotting, real time PCR, and microarray analysis. The purpose of this study is to demonstrate the utility of non-bronchoscopic airway brushing to obtain and study epithelial cells and to encourage others so that we can accelerate our knowledge regarding the role of the epithelium in childhood respiratory disease.

## Background

The use of cytology brushes for the purpose of obtaining respiratory cells from adults for clinical and research purposes is well established. This technique is generally reported to be safe, both in adults with pulmonary disease and in healthy volunteers [1]. Samples are usually obtained under direct vision using a bronchoscope. However, we and others have recently used non-bronchoscopic brushing to sample airway epithelial cells from children [2,3]. This method has several advantages over bronchoscopic brushing as it is simple and quick to perform, and there is no need for a bronchoscope. Although we have now successfully used this method in combination with a variety of cellular and molecular techniques to investigate the role of the epithelium in childhood asthma, initially, very little had been published on the topic. In addition, the difficulty in obtaining target organ tissue from children has meant that most information regarding common childhood diseases such as asthma has been derived from studies performed in adults.

We currently understand little about the molecular mechanisms involved in the pathogenesis of asthma. The airway epithelium is an especially attractive target in which to identify new molecular mechanisms and therapeutic targets because it is critically involved in the development of asthma as the first cell of contact with the environment. This is particularly pertinent since it is likely that dysregulated epithelial repair in childhood asthma is a critical determinant of disease progression in adults. To this end, the available evidence suggests that epithelial fragility and dysfunction as well as accumulation of sub-epithelial fibroblasts and remodelling of the airway wall in asthma can occur early in childhood [4].

The purpose of this report is to summarise our experiences with non-bronchoscopic epithelial brushing techniques and subsequent sample processing. We have detailed the use of cells for gene and protein expression, and for cell culture. The use of primary culture systems has important advantages over the use of immortalized cell lines, and allows functional experiments to be conducted. We aim to encourage the use of these techniques to study normal developmental processes in the lung and the early pathophysiology of respiratory diseases.

## Methods

Children admitted to Princess Margaret Hospital for the purposes of elective surgery for non-respiratory complaints were recruited for this study. Children were usually having minor gastrointestinal or ear, nose and throat surgery. The study was approved by the Princess Margaret Hospital for Children Ethics Committee, and written informed consent was obtained from the parents of the children prior to sampling (see appendix for parent information sheet). The asthmatic and atopic backgrounds of the children were determined by allergen-specific IgE testing and a validated asthma and allergy questionnaire was administered to the parent or legal guardian [5]. Prior to surgery, each child was anaesthetised and intubated. A nylon cytology brush (BC 25105, Olympus, Australia) was used to sample cells from the airway. The plastic sheath protecting the brush was removed and discarded, as retracting the brush into the tightly fitting sheath would dislodge cells. The unprotected brush was inserted directly through the endotracheal tube, advanced until resistance was felt, and rubbed against the epithelial surface to sample cells. The brush was then withdrawn and the tip cut off into 5 ml of culture media (RPMI-1640 containing 10% (v/v) heat inactivated foetal calf serum). This process was repeated at least once.

A sub-group of twenty five of these children was studied to assess how well the technique was tolerated. Respiratory variables were monitored before, during, and after the brushing procedure. Symptoms following the brushing were recorded by contacting the parents within one week of the procedure. Children who underwent non-bronchoscopic brushing (12 male, mean age 10.2, SE 0.71) were compared to 24 control children who were anaesthetised and intubated only for similar procedures (15 male, mean age 10.9, SE 0.76).

### Sample Processing

Epithelial cells were collected into RPMI-1640 containing 10% (v/v) heat inactivated foetal calf serum. Samples were processed immediately. An aliquot of the cell suspension was diluted 1:2 with 0.4% trypan blue, and applied to a haemocytometer. The total number of cells, and the percentage of viable cells, was determined under a light microscope, within 15 minutes of collection. Each sample of cells obtained by non-bronchoscopic brushing was processed to allow for use in multiple investigative techniques; 1 × 10^6 ^cells were used for cell culture or protein extraction, 0.5 × 10^6 ^cells to produce cytospin slides for immunocytochemical studies, and RNA was extracted from the remaining 1 × 10^6 ^cells.

For culture, cells were washed once in RPMI-1640 media and the cell pellet resuspended in bronchial epithelial basal media (BEBM, Clonetics, CA) supplemented with bovine pituitary extract (50 mM), insulin (5 mM), hydrocortisone (0.5 mM), gentamicin (0.001%, v/v), amphotericin B (0.0005%, v/v), retinoic acid (0.1 μM), transferrin (10 mM), triiodothyronine (6.5 μM), epinephrine (6.5 μM) and human recombinant epidermal growth factor (EGF: 0.5 μM). The cells were then seeded into a culture vessel (25 cm^2 ^growth surface area) pre-coated with a mixture of fibronectin, collagen and bovine serum albumin, and maintained at 37°C in a humidified incubator. Twenty-four hours post-isolation, unattached cells were collected. These cells were reseeded into the same culture vessel, with fresh media containing Ultroser G (2% v/v; BioSepra, CA), a serum substitute. The collection and reseeding of viable unattached cells was repeated at both 48 and 72 hours post isolation. Subsequent cultures were fed every second day and were usually passaged every 13–16 days.

Before the remaining cell suspension was used for protein and RNA extraction, and to produce cytospin slides, the macrophages were removed by positive selection: the cell suspension was added to a culture dish that had been previously coated with CD-68 antibody (Dako, Australia). The plate was incubated for 20 minutes (37°C, 5% CO_2_) to allow the macrophages to adhere. The suspended epithelial cells were aspirated from the plate, and the macrophages removed using trypsin (0.25%) for subsequent analysis. The macrophage depleted cell suspension was used to produce cytospins, extract protein, and extract RNA.

Cytospin slides were prepared by centrifuging epithelial cells onto a glass slide in a cytocentrifuge (Hettich). Slides were air dried, fixed in 4% paraformaldehyde for 10 minutes, and then stored at -20°C until required. Immunocytochemical staining of the cytospins was used to confirm the purity of the epithelial sample. Antibodies against cytokeratin (a marker for tissue of epithelial origin), α -smooth muscle actin (a marker of myofibroblasts, myoepithelial cells and smooth muscle cells), smooth muscle myosin (a marker of smooth muscle cells), and vimentin (a marker of mesenchymal cells) were used to confirm epithelial cell phenotype.

Immunocytochemical techniques provide only semi-quantitative data about protein expression, and are not sensitive enough to determine levels of protein expression accurately; therefore protein was extracted for analysis with Western blotting. Protein was extracted from the pelleted epithelial cells by lysing the cells in 200 μl of an SDS extraction buffer (20 mM Tris, 1 mM SDS, 1 mM DTT) in the presence of protease inhibitors (Sigma). A commercial assay (Micro BCA Protein Assay, Pierce Biotechnology) was used according to the manufacturers instructions to determine the concentration of total protein in the cell lysate. For each sample, 50 μg of protein was subjected to 12% SDS-PAGE, and immunoblotted with anti-β-actin antibody. Antibody binding was detected with ECL Plus Western Blotting Detection Reagents (Amersham Biosciences).

Total RNA was extracted from epithelial cells using the QIAGEN RNeasy kit (Vic, Australia). RNA quality and quantity was assessed using the Agilent Bioanalyser (Vic, Australia). RNA was prepared from 18 subjects according to a modified version of the protocol of Baugh et al [6] and hybridised to microarrays. For our first study, gene expression profiles from 9 mild, asymptomatic asthmatics were compared to 9 healthy children for a total of 18 arrays. Expression of genes in cells from asthmatic and healthy children was compared using Affymetrix Human Genome U133 Arrays (HG-U95Av2), which examine the expression of approximately 23,000 genes. Real-time PCR was used to validate the array data for specific genes. Real-time PCR was conducted as previously described [3].

Data was reported as mean (SE) and analysed by independent samples t-test. Significance was taken as p < 0.05. The proportion of basal cells was analysed using analysis of variance (ANOVA) to compare the difference between the three phenotype groups.

## Results

### The procedure is well tolerated

The only significant adverse event reported after the procedure was cough (Table 1). The nursing staff recorded cough in 20% of patients undergoing brushing. Parentally reported cough was higher, with 40% of parents reporting cough in those children undergoing the procedure. Cough persisting longer than 4 hours was recorded in 32% of patients, and 28% had cough persisting to the following day. Despite this, 100% of parents said that if asked by a friend "Should my child participate in this study?" they would answer "yes". Children who underwent non-bronchoscopic brushing were, on average, ventilated for a longer period than the control group (27 vs. 22.3 min), but this was not statistically significant (p = 0.066, table 1). There was no difference in any other clinical variables recorded, including the lowest level of oxygen saturation, and the time spent in recovery.

**Table 1:** Symptoms following non-bronchoscopic brushing. Symptoms following non-bronchoscopic brushing, and observations of respiratory parameters before, during, and after brushing. Cough was the only reported symptom, and all parents said they would recommend the study to a friend.

	Controls mean (SE)	Sampled mean (SE)	p-value
Number of participants	24	25	-
Cough recorded	0%	20% (8%)	0.022
Parental reported cough	-	40% (10%)	-
Cough > 4 h	-	32% (9%)	-
Cough following day	-	28% (46%)	-
Recommend to friend?	-	100% (0%)	-
Length of Ventilation (mins)	22.3 (1.85)	27 (1.68)	0.066
Lowest oxygen saturation	98.6 (0.12)	96.8 (1.02)	0.083
Supplemental oxygen	79% (8%)	80% (8%)	0.944
Highest respiratory rate	22.7 (0.77)	22.2 (0.58)	0.607
Time in recovery (mins)	21.5 (2.22)	18.4 (1.40)	0.246

Symptoms and respiratory variables were compared between asthmatic and non-asthmatic children undergoing non-bronchoscopic brushing (Table 2). The nursing staff did not record cough in any of the asthmatic patients. Parentally reported cough was higher in the asthmatic subjects (43% vs. 39%), but this was not statistically significant (p = 0.863). Parents of asthmatic children also reported a higher level of cough persisting to the following day (43% vs. 22%), however this was not statistically significant (p = 0.322).

**Table 2:** Symptoms following non-bronchoscopic brushing. Comparison of symptoms following non-bronchoscopic brushing, and observations of respiratory parameters before, during, and after brushing in asthmatic and non-asthmatic patients.

	Non-asthma mean (SE)	Asthma mean (SE)	p-value
Number of participants	18	7	-
Cough recorded	28% (11%)	0% (0%)	0.129
Parental reported cough	39% (12%)	43% (20%)	0.863
Cough > 4 h	28% (11%)	43% (20%)	0.489
Cough following day	22% (10%)	43% (20%)	0.322
Recommend to friend?	100% (0%)	100% (0%)	-
Length of Ventilation (mins)	28.9 (1.92)	22.1 (2.86)	0.071
Lowest oxygen saturation	96.9 (1.33)	96.4 (1.46)	0.845
Supplemental oxygen	83% (9%)	71% (18%)	0.524
Highest respiratory rate	22.6 (0.86)	21.0 (0.72)	0.282
Time in recovery (mins)	18.8 (1.73)	17.3 (2.50)	0.644

### Epithelial cell retrieval and processing

**Figure 1:**
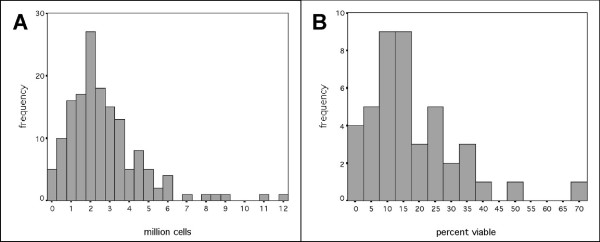
A: Histogram of number of cells sampled in 151 non-bronchoscopic brushings of the paediatric airway. The number of cells retrieved ranged between 0.1 million and 11.8 million (mean 2.7 million). B: Histogram of the percentage of viable cells in 43 non-bronchoscopic brushings of the airway. The mean viability was 17.3%.

**Figure 2:**
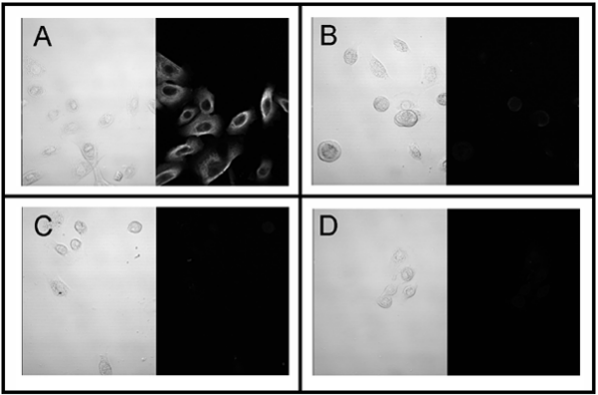
Immunocytochemical confirmation of epithelial cell phenotype. A: Pan-cytokeratin, B: α-smooth muscle actin, C: Smooth muscle myosin, D: Vimentin.

**Figure 3:**
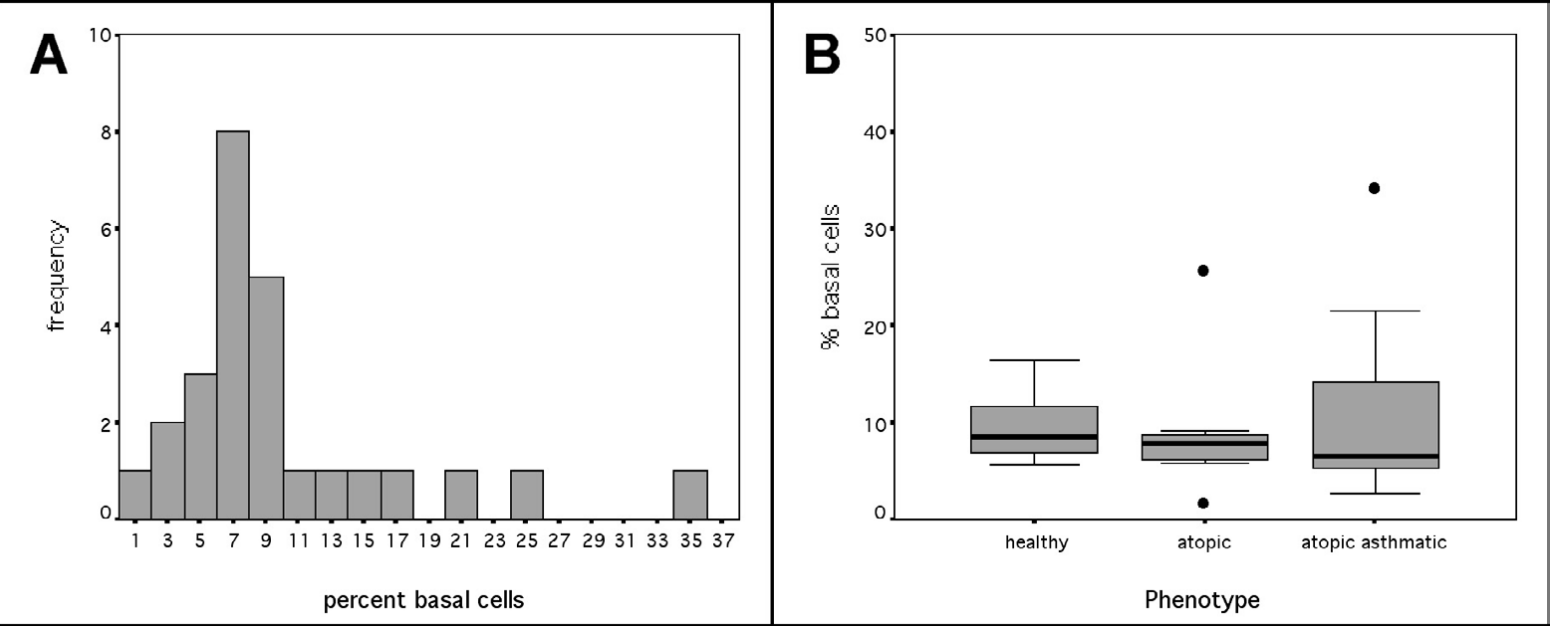
Percentage of basal cells in brushings obtained non-bronchoscopically from the paediatric airway. A: Histogram of the percentage of basal cells in each brushing. On average, 10.04% of cells were basal cells. B: Percentage of basal cells retrieved in brushings from each phenotype. The percentage of basal cells retrieved was not different depending on phenotype.

The mean cell retrieval from 151 brushings was 2.67 × 10^6 ^cells (SE = 0.16 × 10^6^, Figure 1A) and on average, 17.3% of cells were viable (SE = 2.15%, Figure 1B). The number or viability of cells retrieved was not related to asthmatic or atopic status. The epithelial phenotype of the cells was confirmed by immunostaining (Figure 2). To confirm that the population of epithelial cells sampled did not vary between phenotypes, the number of cells expressing the basal cell marker isolectin B4 was determined. The proportion of basal cells was independent of phenotype (Figure 3).

**Figure 4:**
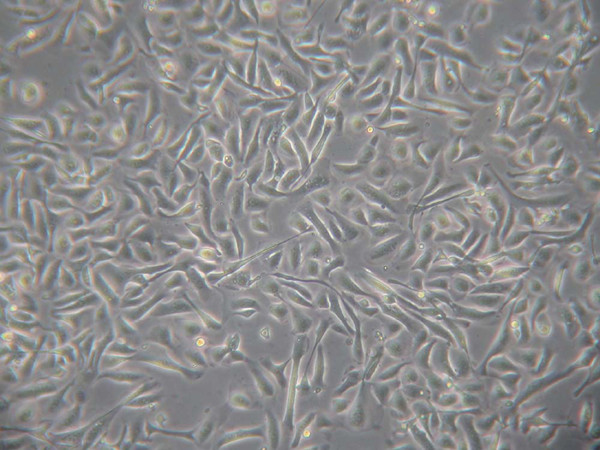
Phase-contrast micrograph of cultured epithelial cells

In our hands, the cell culture success rate is such that 93% of samples exhibit some growth, 80% grow to confluence, and 80% survive a second passage. Initial cultures consisted of a heterogeneous cell population composed of both terminally differentiated epithelial cells as indicated by the presence of cilia, but also non-ciliated epithelial cells. Subsequently passaged cultures were found to solely exhibit the non-ciliated epithelial morphology. No differences in cellular morphology have been observed between cultures established from healthy and asthmatic phenotypes. Established cultures have been successfully grown up to passage 7. When the cells reach 80% confluence in their second passage (Figure 4), they are used for functional studies.

Protein was extracted from the epithelial cells; the average protein yield was 74 μg (SE = 8.79 μg, N = 18) per 1 × 10^6 ^cells. This was sufficient for detection of β-actin using Western blotting, demonstrating the feasibility of determining protein expression from cells obtained by the non-bronchoscopic brushing technique.

**Figure 5:**
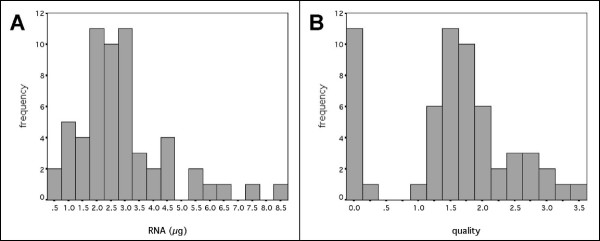
Quantity and quality of RNA extracted from brushings obtained non-bronchoscopically from the paediatric airway. A: Histogram of the amount of RNA extracted. On average, 2.9 μg of RNA was extracted from 2 ml of cell suspension. B: Histogram of the quality of RNA. The ribosomal RNA ratio was used as a measure of RNA quality; on average the RNA quality was 1.52.

RNA extraction yielded on average 2.9 μg of RNA (SE = 0.21, N = 58, Figure 5A), as assayed by Agilent Bioanalyser (Vic, Australia). The ribosomal RNA ratio was used as a measure of RNA quality; the average ratio was 1.52 (SE = 0.12, N = 58, Figure 5B). This was sufficient RNA to use both for microarray analysis (1 μg) and real-time PCR analysis (0.5 μg).

**Figure 6:**
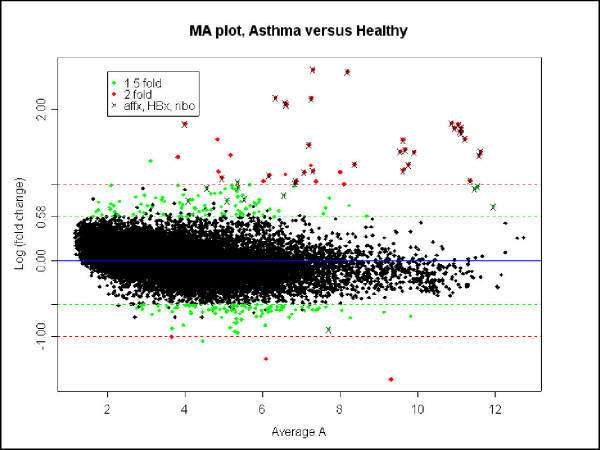
Scatter plot of average gene expression (x axis) vs. log of fold change in gene expression. Those genes which are differentially regulated more than 1.5 fold are highlighted.

Preliminary data from the microarrays showed that gene expression in epithelial cells from asthmatic subjects was significantly different to that in healthy subjects (Figure 6). Expression of genes in the asthmatic cohort ranged from 6-fold up-regulated to 5-fold down-regulated compared to expression in the healthy controls.

## Discussion

We have demonstrated that non-bronchoscopic brushing is safe and well tolerated in children. The main symptom reported following brushing was cough, which was reported more frequently by parents than the nursing staff. The procedure was acceptable to participants, as none of the patients experienced symptoms that would cause the parents not to recommend the study to a friend. Respiratory variables and symptoms in children undergoing brushing were no different in asthmatics compared to non-asthmatics (table 2). The level of cough reported by parents was increased in asthmatic children, however this difference was not significant. The difference was most likely due to increased observation of their child's symptoms, as there was no difference in the level of cough reported by nursing staff in asthmatic and non-asthmatic children.

The non-bronchoscopic brushing technique was able to harvest useful quantities of epithelial cells, with a mean viability of 17.3%. Studies in adult asthmatics, harvesting cells under direct vision, report average viability ranging from 25–30%, with a significantly lower viability in the asthmatic epithelial cells [1,7,8]. In this study, the viability of the epithelial cells was not significantly different in the asthmatics. This may reflect the mild nature of asthma experienced by these children, as previous studies have reported the level of viability is correlated with the severity of the disease [8].

This report outlines a broad range of techniques that can be used to study a single sample of airway epithelium, thus maximising the information obtained from one brushing of the airway. Previous studies conducted in children have detailed only limited use of the sample [2]. We report that it is possible to obtain cells of sufficient quality and quantity to allow investigation using cell culture, immunohistochemistry, western blotting, real-time PCR, and microarray. We have used immunocytochemical staining to examine expression of protein, and sufficient protein can be extracted to perform Western blot analysis. We have cultured the epithelial cells, and can maintain and propagate the cultures successfully over seven passages. Whilst non-bronchoscopic brushings of the paediatric airway are easily and safely obtained, it is important to maximise the amount of information obtained from each brushing. To this end, we have outlined a program of non-bronchoscopic brushing that utilises a wide range of techniques, allowing a broad approach to the study of the airway epithelium in children.

## Conclusion

The purpose of this study was to demonstrate how a single sample of airway epithelial cells obtained by non-bronchoscopic brushing can be used to study gene and protein expression and provides sufficiently viable cells to allow cell cultures to be established for functional analysis. The technique is particularly useful for studying the paediatric airway because it is simple and minimally invasive, and can be used to overcome a major obstacle to our understanding of paediatric respiratory disease namely a paucity of target organ tissue. We have presented data that can be used by other groups to establish similar programs, bench-mark for quality assurance and respond to common questions by human research ethics committees.

## Authors' contributions

CL carried out the protein and RNA analysis, was involved in the cell culture work, and drafted the manuscript. SB designed and carried out the paediatric complications study, and participated in sample processing. AK refined the method for culture of airway epithelial cells. SS conceived of the non-bronchoscopic brushing program. DK established the cell culture program and with SS, participated in the design and coordination of this study, and contributed to the manuscript.

## Appendix

### Parent Information Sheet

#### Does raised exhaled nitric oxide reflect unrecognised airway inflammation in healthy children?

You are being invited to take part in a research study. Before you decide, it is important for you to understand why the research is being done and what it will involve. Please take time to read the following information carefully. Ask us if there is anything that is not clear or if you would like more information. Take time to decide whether or not you wish to take part.

GENERAL INFORMATION:

Nitric oxide (NO) is a molecule that is involved in many physiological processes in the body including inflammation. NO is detectable in exhaled breath (eNO) and is raised in asthmatics. Many studies have demonstrated that exhaled NO might be a useful marker of airway inflammation in asthma thus aiding diagnosis and monitoring of disease activity. However, children who respond to skin allergy tests (atopic) and who do not have any respiratory symptoms also have raised eNO. We do not know why eNO is raised in healthy atopic children but it may also be due to inflammation in the airways that is not presently causing respiratory problems. If we can determine what causes raised eNO in healthy atopic children we will better understand how this test will help us monitor airway disease.

"What will happen if I agree for my child to take part?"

The present study will use standard diagnostic techniques to investigate whether inflammation that might not otherwise be recognised is the cause of raised NO levels in the breath of children with atopy. In order to study this issue we will recruit children, WITH and WITHOUT ATOPY, who are at Princess Margaret Hospital for day surgery.

We will use the following strategy:

• You will be asked to complete a questionnaire about your child's symptoms of allergy and asthma and medications that may effect eNO levels.

• If your child is old enough they will be asked to blow into a machine to determine their eNO levels. This may occur either before the operation or at the time of their next appointment.

• During surgery we will obtain a brushing of the surface of the windpipe.

• A blood sample will be taken for allergy testing to common allergens. It will also be used for gene testing for NO genes and asthma genes.

Measurements we will make:

1. Prior to surgery

**Exhaled nitric oxide **– To measure eNO we will ask your child to take a deep breath in and blow into a machine while trying to maintain a constant expiratory flow.

2. During surgery

**Brushings **– To collect cells from the wall of the airways we will pass a fine brush through the larynx (voice box) into the windpipe and rub along the wall of the windpipe a few centimetres below the vocal cords. If you wish, we will demonstrate the techniques and equipment used before you decide to go ahead.

**Blood sample **– Blood will be collected once your child is asleep.

Risks:

The brushing is a simple test and takes less than 5 minutes to complete. In adults it is performed without an anaesthetic. The test will not be carried out if your child's anaesthetist or surgeon believes the test will interfere with your child's treatment. We have routinely performed hundreds of these tests without incident. A dry cough is the only adverse symptom reported, it seems to occur in approximately half of the children involved in our study.

Benefits:

We will be able to provide you with information regarding the allergic status of your child.

All information that is collected about your child during the course of the research will be kept strictly confidential. Any information about your child that leaves the hospital will have your/his/her name and address removed so that you/he/she cannot be recognised from it.

It is up to you to decide whether or not to take part. If you decide to take part you will be given this information sheet to keep and be asked to sign a consent form. If you decide to take part you are still free to withdraw at any time and without giving a reason. This will not affect the standard of care your child receives.

If you have any complaints about any aspect of the study you can contact the Executive Director Medical Services of PMH (Dr Geoff Masters) on 9340 1550

Thank you for reading this information sheet.

If you have any further questions with regard to this study they can be discussed with

Dr Stephen Stick (Telephone 9340 8830)

Dr Scott Burgess (Hospital switch board – Telephone 9340 8222 page 2025 at any time)
